# Band Ratios Matrix Transformation (BRMT): A Sedimentary Lithology Mapping Approach Using ASTER Satellite Sensor

**DOI:** 10.3390/s18103213

**Published:** 2018-09-23

**Authors:** Ghasem Askari, Amin Beiranvand Pour, Biswajeet Pradhan, Mehdi Sarfi, Fatemeh Nazemnejad

**Affiliations:** 1 School of Earth Sciences, Damghan University, Damghan 3671641167, Iran; gh.askari@du.ac.ir (G.A.); m.sarfi@du.ac.ir (M.S.); f.nazemnezhad1995@gmail.com (F.N.); 2 Korea Polar Research Institute (KOPRI), Songdomirae-ro, Yeonsu-gu, Incheon 21990, Korea; beiranvand.amin80@gmail.com; 3 Centre for Advanced Modelling and Geospatial Information Systems (CAMGIS), Faculty of Engineering and Information Technology, University of Technology Sydney, Ultimo 2007, Australia; 4 Department of Energy and Mineral Resources Engineering, Choongmu-gwan, Sejong University, 209 Neungdong-ro Gwangjin-gu, Seoul 05006, Korea

**Keywords:** Band Ratios Matrix Transformation (BRMT), lithostratigraphy mapping, ASTER, Deh-Molla, sedimentary rocks

## Abstract

Remote sensing imagery has become an operative and applicable tool for the preparation of geological maps by reducing the costs and increasing the precision. In this study, ASTER satellite remote sensing data were used to extract lithological information of Deh-Molla sedimentary succession, which is located in the southwest of Shahrood city, Semnan Province, North Iran. A robust and effective approach named Band Ratio Matrix Transformation (BRMT) was developed to characterize and discriminate the boundary of sedimentary rock formations in Deh-Molla region. The analysis was based on the forward and continuous division of the visible-near infrared (VNIR) and the shortwave infrared (SWIR) spectral bands of ASTER with subsequent application of principal component analysis (PCA) for producing new transform datasets. The approach was implemented to ASTER spectral band ratios for mapping dominated mineral assemblages in the study area. Quartz, carbonate, and Al, Fe, Mg –OH bearing-altered minerals such as kaolinite, alunite, chlorite and mica were appropriately mapped using the BRMT approach. The results match well with geology map of the study area, fieldwork data and laboratory analysis. Accuracy assessment of the mapping result represents a reasonable kappa coefficient (0.70%) and appropriate overall accuracy (74.64%), which verified the robustness of the BRMT approach. This approach has great potential and capability for mapping sedimentary succession with diverse local–geological–physical characteristics around the world.

## 1. Introduction

Preparation of geological maps and mineral exploration using conventional fieldwork investigations are time-consuming and expensive for geologists and mining companies [1,2,3]. Remote sensing plays a key role in geological mapping and mineral exploration, especially in complex, remote and inaccessible areas [4,5,6,7,8,9]. For the preparation of lithological maps using multi-spectral satellite imagery, numerous image processing methods have been developed and used by researchers [10,11]. Spectral indices and band ratios are the most common image processing methods for spectral enhancement to map lithological units [12,13,14,15,16,17]. 

Sedimentary rocks cover 75% of the Earth’s surface, but a few image processing methods have been proposed for mapping sedimentary lithological units [18,19,20,21]. The lack of specialized image processing method for mapping sedimentary rock units motivated us to develop a robust and easily applicable method to map the sedimentary strata. Some of the significant studies that used ASTER spectral bands for sedimentary rock mapping are summarized as follows. Ninomiya and Fu [18] proposed quartz (QI), carbonate (CI), and silica (SI) indices for the identification and separation of sedimentary rock units such as quartzite, dolomite and limestone, which mainly use the TIR bands of the ASTER. Öztan and Süzen [20] presented a method in order to identify the Ankara gypsum units. This method is based on a combination of decorrelation stretch, band ratio, feature-oriented principal component Analysis (FOPCA), and thermal indices such as CI, QI, and SI. Several Red-Green-Blue (RGB) composites of the band ratios 4/6, 4/9 and 8/6 along with 4/(6 + 8) and 9/(4 + 8) were used to identify gypsum units. Finally, the Sulfate Index (SI) = (10 × 12)/(11 × 11) was developed by the authors for mapping Ankara gypsum units.

Zadeh and Tangestani [21] mapped the sedimentary units (sandstone, tuff, conglomerate, siltstone and limestone) of the southeast of Iran in Kerman province using thermal bands of ASTER. Spectral Angel Mapper (SAM) method was used to prepare the geological map of the area. The Black Hatat area of eastern Oman was studied by Rajendran et al. [22] using Band ratio of 7 + 9/8 for limestone, 6 + 8/7 for dolomite, 2/1 for Fe^3+^ rich mafic rocks and 6/8 for rocks rich in Quartz (such as shale, schist, and sandstone). Pour et al. [8] developed several ASTER band ratios such as Fe-Minerals Index (Fe-MI) = (4/3) × (2/1), Al-OH-bearing alteration Minerals Index (Al–OH-MI) = (5 × 7)/(6 × 6) and Fe, Mg–OH-bearing alteration Minerals Index (Fe, Mg–OH-MI) = (7 × 9)/(8 × 8) for mapping sedimentary sequences in the Northern Victoria Land, Antarctica.

Accordingly, band ratios and PCA have been widely used for identifying rock and mineral units. Band ratios mostly rely on the absorption and reflection wavelengths of minerals, which have led to the establishment of several spectral indices for specific minerals or rocks. It is a threshold dependent technique. The principal component analysis (PCA) is a well-known method for lithological and alteration mapping in metallogenic provinces [14,15,23], which is a scene dependent method. Therefore, these widely used techniques for lithological–mineralogical mapping may have different outputs for a scene with different size and threshold. It results in low accuracy discrimination between lithological units, especially for sedimentary rock boundaries. Moreover, the application of band ratios for lithological mapping may contain some false results due to the spectral interference of the minerals with similar absorption and reflection properties. Hence, if only band ratio is used for producing a geological map of a study region, some of the spectral interference can result in lithological boundary discrimination with low accuracy.

**Figure 1:**
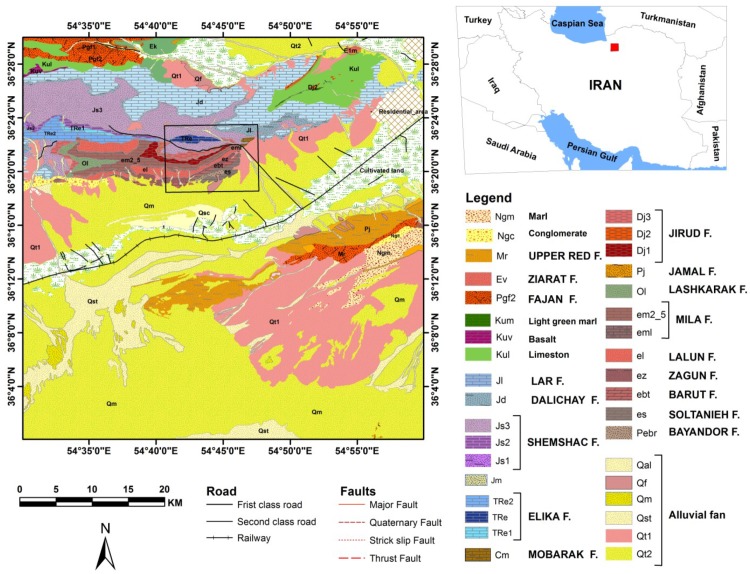
Geographical location of the Deh-Molla inside Shahrood geological map (scale 1:50000) and its location in North of Iran (red cube). Black rectangle shows the subset of the Deh-Molla sedimentary succession in the Shahrood geological map.

To overcome these problems, Band Ratios Matrix Transformation (BRMT) was developed in this study. This algorithm depends on the type of rocks in the selected region of interest. It is because of consideration of all possible band ratios for extraction of the main components in the scene. A forward and non-repetitive Band Ratios (BR) matrix of VNIR and SWIR ASTER spectral bands was constructed and, subsequently, PCA transformation was implemented to the matrix. In this study, BRMT approach was established for sedimentary lithological mapping. The Deh-Molla sedimentary succession was selected for testing the BRMT method, which is located in the southwest of Shahrood city, Semnan Province, North Iran (Figure 1). This region has arid to semi-arid climate. Yearly precipitation is very low; hence, the surface is well-exposed due to sparse and non-existent vegetation cover. The Deh-Molla area hosts main stratigraphic units of the Alborz Range such as Fasham, Deh-Sufiyan and Deh-Molla. Moreover, stratigraphic rock units are well-exposed, and the region is easy to access through Damghan-Shahrood highway. Therefore, this area could be considered as a suitable case study area for remote sensing analysis especially for testing the BRMT technique that developed in this imagery. The objectives of this study were: (i) to develop the mathematics performance of BRMT algorithm and its effects on spectral characteristics of the input bands; (ii) to test and apply BRMT algorithm to the Deh-Molla sedimentary stratigraphic units from the imagery; and (iii) to compare the results of BRMT algorithm with well-established ASTER mineralogical/lithological indices and fieldwork and laboratory analysis.

## 2. Geological Setting and Stratigraphy of the Study Area 

**Figure 2:**
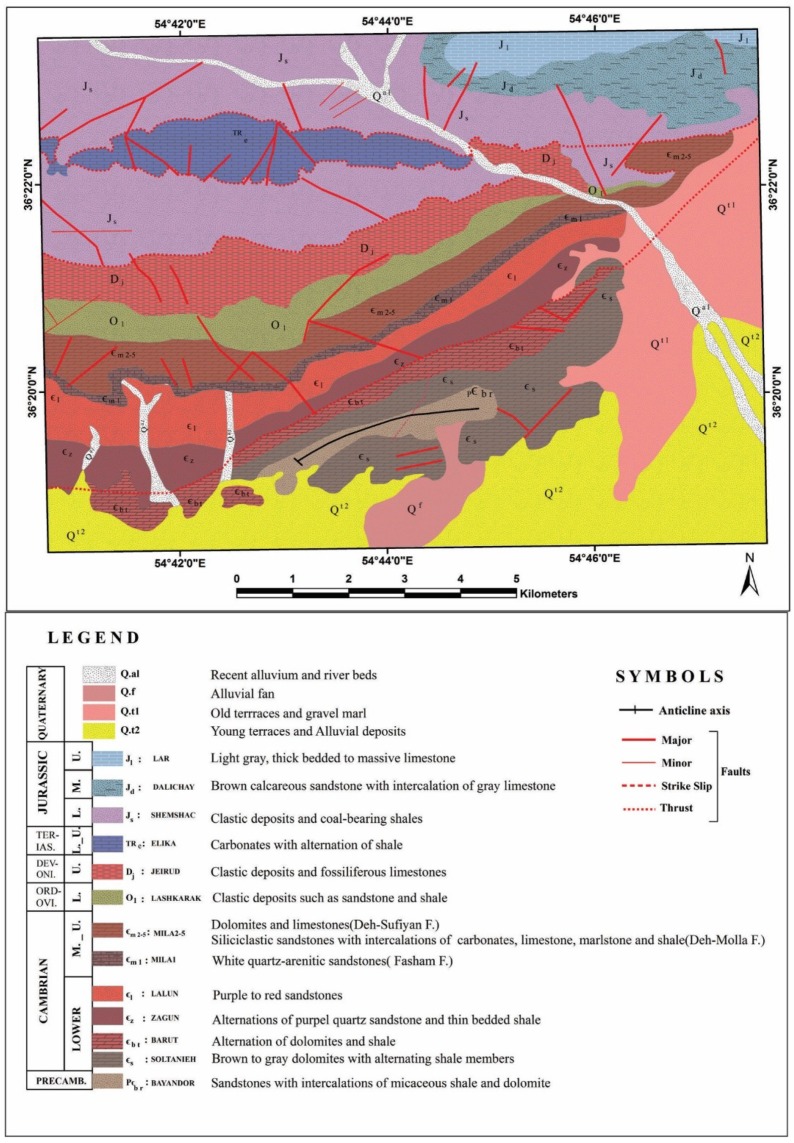
Geological map of the Deh-Molla illustrates the sedimentary Formations in the area of study.

The Alborz Mountains of northern Iran extend about 2000 km along the southern margin of the Caspian Sea from Azerbaijan Province (west Iran) to western Afghanistan. The stratigraphic succession of the Alborz has been outlined to the central and eastern Alborz stratigraphic rock units. Their thickness is approximately 11–13 km and shows Pre-Cambrian to Phanerozoic age [24,25,26]. The study area is located near Deh-Molla Village, approximately 15 km southwest of the Shahrood city, Semnan Province, north Iran (Figure 1). The Deh-Molla stratigraphic section is part of the Central Alborz, which is located close to the boundary of eastern Alborz Range. According to geologic map of the Shahrood (scale 1:100000), sedimentary succession of this area started by the Bayandor Formation and reached to the Jurassic strata of the Shemshak Formation (Figure 2) [27].

The Bayandor Formation is the oldest exposed rock units of the Deh-Molla, consisting of 120 m of sandstones with intercalations of micaceous shale and dolomite. The Soltanieh Formation mainly consists of brown to gray dolomites with alternating shale members (latest Proterozoic to Early Cambrian) [26]. The Barut Formation composed of an alternation of dolomites and shale (Early Cambrian). The overlying Zagun Formation in the Deh-Molla section is not well exposed and consists of about 100 m of shales and sandstones. The Lalun Formation mainly consists of purple to red sandstones (Late Early Cambrian) (Figure 2). In the Deh-Molla section, it has about 400 m thickness.

The Fasham Formation is a newly introduced rock unit by Geyer et al. [25]. Type section of this rock unit is also located in the Deh-Molla section. Fasham Formation consists of 60–100 m of white quartz-arenitic sandstones [28]. This stratigraphic shift is a consequence of an unconformity recognized at the base of this quartzarenite unit. It is traditionally called Mila Formation sensu [26], which was subdivided into five members. Mila Group comprises Fasham, Deh-Sufyian, Deh-Molla, and Lashkarak Formations. The Deh-Sufyian Formation could be subdivided into two units that are in accordance with the informal “Member 1” and “Member 2” of the traditional Mila Formation. This formation predominantly composed of carbonate rocks. The basal unit mainly composed of dolostones, which is followed by limestones of the upper unit (Middle Cambrian) [25]. Lithologically the Deh-Molla Formation consists of two recognizable units that correspond to “Member 3” and “Member 4” of the Mila Formation. The lower unit dominated by siliciclastic sandstones with intercalations of carbonates interlayers. The upper unit consists of limestone, marlstone and shale [25]. 

Ordovician strata of the Alborz Mountain include Member 5 of the Mila, Lashkarak and Gheli Formations and unnamed Formation [25,26,29]. In this study, we preferred to use the term of the Lashkarak Formation for Lower Ordovician deposits of the Deh-Molla area. Lashkarak Formation is mainly composed of clastic deposits such as sandstone and shale. Some narrow igneous and limestone intercalations are also intermittently observed [30]. The Jeirud Formation overlay the Lashkarak deposits with a significant unconformable transition (Figure 2). The Jeirud at the base mainly composed of clastic deposits and towards to up-section contains fossiliferous limestones (Upper Devonian). Mesozoic rock units of the Deh-Molla area include Elika, Shemshak, Dalichay, and Lar Formations (see Figure 2). Carbonates of the Elika Formation (Early to Middle Triassic) are surrounded by Shemshak Formation clastic deposits. The Late Triassic Early Jurassic sandstones and coal-bearing shales of the Shemshak Formation with a thickness of more than 1100 m are one of the main stratigraphic rock units of the Deh-Molla area. These rock units with some coal-bearing strata are considerable for economic exploitation and have some coal mines. Quaternary alluvium deposits covered the river beds and foot-hill parts in the Deh-Molla area. Lithological compositions of the sedimentary formations reported in the Deh-Molla region are summarized in Table 1.

## 3. Materials and Methods

### 3.1. ASTER Data and Pre-Processing Analysis

The Advanced Spaceborne Thermal Emission and Reflection Radiometer (ASTER) sensor consists of three separate instrument subsystems with a total of 14 bands valuable for lithological–mineralogical mapping, including: (i) three bands in the visible-near infrared (VNIR) subsystem from 0.52 to 0.86 μm with a 15 m spatial resolution, which are suitable for iron oxide/hydroxide identification; (ii) six bands in the shortwave infrared (SWIR) subsystem from 1.60 to 2.43 μm with a 30 m spatial resolution, which are useful for clay mineral discrimination; and (iii) five bands in the thermal infrared (TIR) subsystem from 8.12 to 11.65 μm with a 90 m spatial resolution, which can be used for mapping silicate and carbonate minerals [31,32]. ASTER swath width is 60 km that each individual scene is cut to a 60 × 60 km^2^ area [33].

The ASTER Level 1 T (Precision Terrain Corrected Registered At-Sensor Radiance (AST_L1T)) data acquired on 5 August 2007 was obtained from NASA Earth data center (https://earthdata.nasa.gov/about/daacs/daac-lpdaac) for this study. AST_L1T contains calibrated at-sensor radiance, which corresponds with the ASTER Level 1B (AST_L1B), which has been geometrically corrected and rotated to a north-up UTM projection with WGS84 datum. The reflectance of VNIR and SWIR bands of the datasets has been corrected by incorporating the atmospheric condition through SCP plugin (QGIS 2.18) software [34]. Moreover, Crosstalk correction [35] was also applied to ASTER data. The VNIR reflectance data was resized to 30-m resolution of SWIR bands for further spatial and spectral image processing. It can help to maintain the maximum spectral properties of earth surface materials in the SWIR region, which conveys key spectral information for a wide variety of rock-forming minerals [36].

### 3.2. Fieldwork, Laboratory Data and Accuracy Assessment 

For verification of the image processing analysis, the results were compared with the published geology map of the Shahrood (scale 1:100000) [27] prepared by geological survey of Iran, and fieldwork observation that was carried out on 15 August 2017 in the study area. Geological locations were measured by a GPS (Garmin eTrix 10, Nanjing Sifang Mapping Equipment Ltd., Jiangsu, China) survey. Ground photos were taken of the geomorphology and sedimentary rock units. Samples for laboratory studies were collected through a systematic sampling of fresh and surface-weathered sides of the representative sedimentary lithologies. The XRD analyses were implemented on bulk powder using an X-ray diffractometer, Advance-D8 XRD Bruker model, at Central Laboratory of Damghan University, Damghan, Iran. Moreover, several thin sections of the sedimentary rocks were provided for optical microscopy analyses of sedimentary sequences exposed in the study area.

The accuracy of image classification is most often reported as error matrix include of user’s accuracy (UA), producer’s accuracy (PA), and kappa coefficient (*κ*) [37]. UA is computed using the number of correctly classified pixels to the total number of pixels assigned to a particular class. PA defines the number of pixels correctly classified in a particular class as a percentage of the total number of pixels actually belonging to that class in the image. The Kappa Coefficient is generated from a statistical test to evaluate the accuracy of a classification [38]. Kappa essentially evaluates how well the classification performed as compared to just randomly assigning values. The Kappa Coefficient can range from −1 to 1. A value of 0 indicated that the classification is no better than a random classification. A negative number indicates the classification is significantly worse than random. A value close to 1 indicates that the classification is significantly better than random [39,40]. In this analysis, accuracy assessment was performed by comparing the map produced by remote sensing analysis and the reference geological map of the study area. For this purpose, the maps have been registered geometrically to each other using accurate GPS reading in the study area. Additionally, a similar classification scheme and spatial detail were used for this assessment.

### 3.3. Band Ratios Matrix Transformation (BRMT)

A forward and non-repetitive Band Ratios (BR) matrix of VNIR and SWIR ASTER spectral bands was constructed and. subsequently, PCA transformation was implemented to the matrix as a vector (see Equation (1)). Vectorization of the matrix is necessary for PCA implementation.

(1) $$M_{m}^{T}=PCA[\begin{array}{c} n_{1\;\;}\;\;\;\;\;n_{2\;\;}\;\;\;\;\;n_{3\;\;}\;\;\;\;\;n_{4\;\;}\;\;\;\;\;n_{5\;\;}\;\;\;\;\;n_{6\;\;}\;\;\;\;n_{7\;\;}\;\;\;\;\;n_{8\;\;}\\ n_{9\;}\;\;\;\;\;n_{10\;}\;\;\;\;\;n_{11\;}\;\;\;\;\;n_{12\;}\;\;\;\;\;n_{13\;}\;\;\;\;\;n_{14\;}\;\;\;\;n_{15\;}\;\;\\ n_{16\;}\;\;\;\;\;n_{17\;}\;\;\;\;\;n_{18\;}\;\;\;\;\;n_{19\;}\;\;\;\;\;n_{20\;}\;\;\;\;\;n_{21\;}\\ n_{22\;}\;\;\;\;\;n_{23\;}\;\;\;\;\;n_{j\;\;}\;\;\;\;\;n_{25\;}\;\;\;\;\;n_{26\;}\;\\ n_{27\;}\;\;\;\;\;n_{28\;}\;\;\;\;\;n_{29\;}\;\;\;\;\;n_{30\;}\;\\ n_{31\;}\;\;\;\;\;n_{32\;}\;\;\;\;\;n_{33\;}\;\\ n_{34\;}\;\;\;\;\;n_{35\;}\;\\ n_{36\;}\; \end{array}]=PCA\left[\begin{array}{c} n_{1}\\ n_{2}\\ n_{3}\\ .\\ .\\ .\\ .\\ n_{36} \end{array}\right]=\left[\begin{array}{c} P_{1}\\ P_{2}\\ P_{3}\\ .\\ .\\ .\\ .\\ P_{36} \end{array}\right]$$

where M is the band ratios matrix (BRM), T is the transformation of the matrix using PCA method, and m indicates the number of input bands (assumed to be nine in Equation (1)), which is associated to a total number of VNIR and SWIR bands of ASTER datasets. P1–P36 are resultant principal components, and nj is the band ratio used for the construction of BRM in which the identity number (j) belongs to the set of J = {1, 2, 3, …, j, …, 36}.

Band ratio matrix (BRM) is a set of band ratios constructed by the forward and backward division of VNIR and SWIR bands, mutually (one by one). In the forward condition, the band identity number of numerators is smaller than denominators, i.e., the set of ratios *b*1/*b*2, *b*1/*b*3, *b*1/*b*4, etc.; b2/b3, *b*2/*b*4, *b*2/*b*5, etc.; *b*3/*b*4, *b*3/*b*5, *b*3/*b*6, etc.; and so on build the BRM. In the backward condition, the band identity number of numerators is larger than the denominator, i.e., the ratios *b*9/*b*8, *b*9/*b*7, *b*9/*b*6, etc.; *b*8/*b*7, *b*8/*b*6, *b*8/*b*5, etc.; *b*6/*b*5, *b*6/*b*4, *b*6/*b*3, etc.; and so on build the BRM. The following matrices in Equations (2) and (3) represent the forward and backward BRM, respectively. The matrix elements are the band ratios of ASTER SWIR and VNIR reflectance bands.

(2) $$Forward=(\begin{array}{c} \frac{b1}{b2}\;\;\;\;\frac{b1}{b3}\;\;\;\;\frac{b1}{b4}\;\;\;\;\frac{b1}{b5}\;\;\;\;\frac{b1}{b6}\;\;\;\;\frac{b1}{b7}\;\;\;\frac{b1}{b8}\;\;\;\;\frac{b1}{b9}\\ \frac{b2}{b3}\;\;\;\;\frac{b2}{b4}\;\;\;\;\frac{b2}{b5}\;\;\;\;\frac{b2}{b6}\;\;\;\;\frac{b2}{b7}\;\;\;\;\frac{b2}{b8}\;\;\;\frac{b2}{b9}\\ \frac{b3}{b4}\;\;\;\;\frac{b3}{b5}\;\;\;\;\frac{b3}{b6}\;\;\;\;\frac{b3}{b7}\;\;\;\;\frac{b3}{b8}\;\;\;\;\frac{b3}{b9}\\ \frac{b4}{b5}\;\;\;\;\frac{b4}{b6}\;\;\;\;\frac{b4}{b7}\;\;\;\;\frac{b4}{b8}\;\;\;\;\frac{b4}{b9}\\ \frac{b5}{b6}\;\;\;\;\frac{b5}{b7}\;\;\;\;\frac{b5}{b8}\;\;\;\;\frac{b5}{b9}\\ \frac{b6}{b7}\;\;\;\;\frac{b6}{b8}\;\;\;\;\frac{b6}{b9}\\ \frac{b7}{b8}\;\;\;\;\frac{b7}{b9}\\ \frac{b8}{b9} \end{array})$$

(3) $$Backward=(\begin{array}{c} \frac{b9}{b1}\;\;\;\;\frac{b9}{b2}\;\;\;\;\frac{b9}{b3}\;\;\;\;\frac{b9}{b4}\;\;\;\;\frac{b9}{b5}\;\;\;\;\frac{b9}{b6}\;\;\;\frac{b9}{b7}\;\;\;\;\frac{b9}{b8}\\ \frac{b8}{b1}\;\;\;\;\frac{b8}{b2}\;\;\;\;\frac{b8}{b3}\;\;\;\;\frac{b8}{b4}\;\;\;\;\frac{b8}{b5}\;\;\;\;\frac{b8}{b6}\;\;\;\frac{b8}{b7}\\ \frac{b7}{b1}\;\;\;\;\frac{b7}{b2}\;\;\;\;\frac{b7}{b3}\;\;\;\;\frac{b7}{b4}\;\;\;\;\frac{b7}{b5}\;\;\;\;\frac{b7}{b6}\\ \frac{b6}{b1}\;\;\;\;\frac{b6}{b2}\;\;\;\;\frac{b6}{b3}\;\;\;\;\frac{b6}{b4}\;\;\;\;\frac{b6}{b5}\\ \frac{b5}{b1}\;\;\;\;\frac{b5}{b2}\;\;\;\;\frac{b5}{b3}\;\;\;\;\frac{b5}{b4}\\ \frac{b4}{b1}\;\;\;\;\frac{b4}{b2}\;\;\;\;\frac{b4}{b3}\\ \frac{b3}{b1}\;\;\;\;\frac{b3}{b2}\\ \frac{b2}{b1} \end{array})$$

BRM could be reconstructed for vide verity of multi-spectral remotely sensed datasets by manipulating the matrix elements. Equation (4) is proposed to calculate the total number of the matrix element, Sm, as follows:

(4) $$S_{m}=\sum_{i=1}^{m-1}\left[\begin{array}{c} r_{1}=\left(m-1\right)\\ r_{2}=\left(m-2\right)\\ r_{3}=\left(m-3\right)\\ .\\ .\\ .\\ r_{i}=\left(m-i\right)\\ .\\ .\\ .\\ r_{m-1}=m-\left(m-1\right) \end{array}\right]=\frac{m^{2}-m}{2}\;$$

where m identifies the number of VNIR and SWIR bands, *ri* is the sum (total number) of elements in *i*th row of the matrix, and *m*-1 is the number of rows in the BRM. For example, in this study, for ASTER VNIR + SWIR bands, the S_m_ and *ri* were calculated using Equation (4) as follows:

$$\;S_{m}=\overset{7}{\underset{i=1}{\sum }}\left[\begin{array}{c} r_{1}=\left(9-1\right)=8\\ r_{2}=\left(9-2\right)=7\\ r_{3}=\left(9-3\right)=6\\ r_{4}=\left(9-4\right)=5\\ r_{5}=\left(9-5\right)=4\\ r_{6}=\left(9-6\right)=3\\ r_{7}=\left(9-7\right)=2\\ r_{8}=\left(9-8\right)=1 \end{array}\right]=\frac{81-9}{2}=36\;$$

The above matrices contain 36 band ratios and hold spectral properties of rock constituents in the image. This initial information is generally correlated and requires to be translated in new format. PCA is a transformation method for reducing intercorrelated variables into a few variables called principal components (PCs). This transformation is defined in such a way that the first principal component has the largest possible variance and each succeeding component has a lower variance [41,42]. Correspondingly, BRM forward and backward matrices were converted into new uncorrelated variables (components or bands herein as BT bands) using PCA analysis, which are called *f^t^* and *b^t^* for forward and backward conditions, respectively (Equations (5) and (6)).

(5) $$\;f^{t}=\left[\;Pf_{1}\;\;\;Pf_{2}\;\;\;Pf_{3}\;\;\;.\;\;\;.\;\;\;.\;\;\;Pf_{i}\;\;\;.\;\;\;.\;\;\;.\;\;\;Pf_{n}\;\right]\;$$

(6) $$\;b^{t}=\left[Pb_{1}\;\;\;Pb_{2}\;\;\;Pb_{3}\;\;\;.\;\;\;.\;\;\;.\;\;\;Pb_{i}\;\;\;.\;\;\;.\;\;\;.\;\;\;Pb_{n}\right]\;$$

where *pf_i_* and *pb_i_* are the *i*th principal component of the forward and backward matrices, and *n* is equal to S_m_. 

Statistical result of PCA transformation (eigenvalues and eigenvector loadings) is effective to specify most variant components of *f^t^* and *b^t^*. Equation (7) was used to calculate the variance percent of the components as follow:

(7) $$\;\%V_{i}=\frac{e_{i}}{\sum_{i=1}^{n}e_{i}}\times 100\;\;$$

where *ei* indicates eigenvectors in ith component, *i* presents identity number of the components, and n is defined above. Most variant eigenvalues of the forward BRMT are reported in Table 1.

Magnitude and sign of the eigenvector loadings define the contribution of band ratios in each component [43,44]. Equation (8) shows the Pearson correlation coefficient between band ratios (of BRM) and the resulting components, i.e., *f^t^* and *b^t^* vectors.

(8) $$\;{\left[r\left(p_{k},\;n_{j}\right)\right]}_{j,k=1}^{s_{m}}=\frac{N\sum_{l=1}^{N}p_{l}n_{l}-\left[\sum_{l=1}^{N}p_{l}\times \sum_{l=1}^{N}n_{l}\right]}{\sqrt{\left[N\sum_{l=1}^{N}p_{l}^{2}-{\left(\sum_{l=1}^{N}n\right)}^{2}\right]\times \left[N\sum_{l=1}^{N}p_{l}^{2}-{\left(\sum_{l=1}^{N}n\right)}^{2}\right]}}\;$$

where *r* is the set of correlations between *j*th band ratio (*n_j_*) and *k*th resulting principal components (*p_j_*), *N* indicates the total number of pixels in *p* (principal components) and *n* (band ratio) images, and *l* presents the pixel identity number. 

Equation (9) is used to define the average correlation of band ratio vector (*n*1–*n*36) in each principal component. This equation calculates the negative and positive averages correlations separately.

(9) $$\;{\left[{\bar{r}}_{k}\right]}_{k=1}^{s_{m}}=\mp \frac{\sum_{j=1}^{m}\left|r\left(p_{k},n_{j}\right)\right|>0.1}{e}\;$$

where *r_k_* presents the absolute correlation coefficient of the *k*th principal component (*p_k_*,) and the associated band ratio vector (*n_j_*), “||” is the absolute symbol, *e* denotes the total number of band ratios candidates showing meaningful absolute correlation i.e., |*r*(*p_k_, n_j_*)| > 0.1, and ± symbol is used to switch between negative and positive correlations. The average correlation of band ratio candidates for *p*_1_–*p*_12_ is presented in Table 2 for this analysis. 

Equation (10) is used to define the average contribution of each band ratio in the construction of principal component vector. This conditional equation also calculates the negative and positive averages.

(10) $$\;{\left[{\bar{r}}_{j}\right]}_{j=1}^{s_{m}}=\pm \frac{\sum_{k=1}^{s_{m}}(\left|r\left(p_{k},n_{j}\right)\right|>0.1)}{h}\;$$

where *r_j_* represents the absolute correlation coefficient of the *j*th band ratio (*n_j_*) and the associated principal component vector (*p_k_*), *h* denotes the total number of components showing meaningful absolute correlation i.e., |*r*(*p_k_*,*n_j_*)| > 0.1. The negative and positive correlations for *n*1–*n*36 band ratios are presented in Table 3.

To calculate the contribution percentage of the band ratios, Equation (11) is proposed, which is a type of normalization strategy.

(11) $$\;\%C_{j}=\pm \frac{\left|r\left(p_{k},n_{j}\right)\right|}{\sum_{k=1}^{s_{m}}(\left|r\left(p_{k},n_{j}\right)\right|}\times 100\;$$

where %*C_j_* indicates the contribution of *j*th band ratio in the construction of resulting principal components, i.e., *f_t_ or b_t_.* Table 4 shows the contribution percentage of band ratios *n*1–*n*36 for building the resultant principal components. 

Furthermore, Equation (8) is used to calculate the correlation coefficients among 36 band ratios, which the highly correlated band ratios (*r* > 0.90) are defined and listed in Table 5. Considering statistical results derived from Table 1, Table 2, Table 3, Table 4 and Table 5 for the selected sub-scene of the study area, it is evident that some of the BRMT bands show the similar manifestation of sedimentary formations due to high negative correlation, low contribution percentage and high correlation of the band ratios. However, a small number of the BRMT bands specifically map the sedimentary formations in the study area. Table 6 shows BRMT bands with specific manifestation (dark or bright pixels) for the sedimentary formations exposed in the Deh-Molla area. Subsequently, BRMT bands (abbreviated as BT bands) contain specific spectral information were selected as input datasets for post classification using rule image classifier tool. BT1, BT2, BT3, BT4, BT5, BT6, BT7, BT11, BT13, BT18 and BT32 were selected as rule images (input BRMT bands) (Table 7). The threshold value of 0.750 with maximum value option was applied for running the rule image classifier tool. 

## 4. Results

### 4.1. Fieldwork and Laboratory Analysis Results

**Figure 3:**
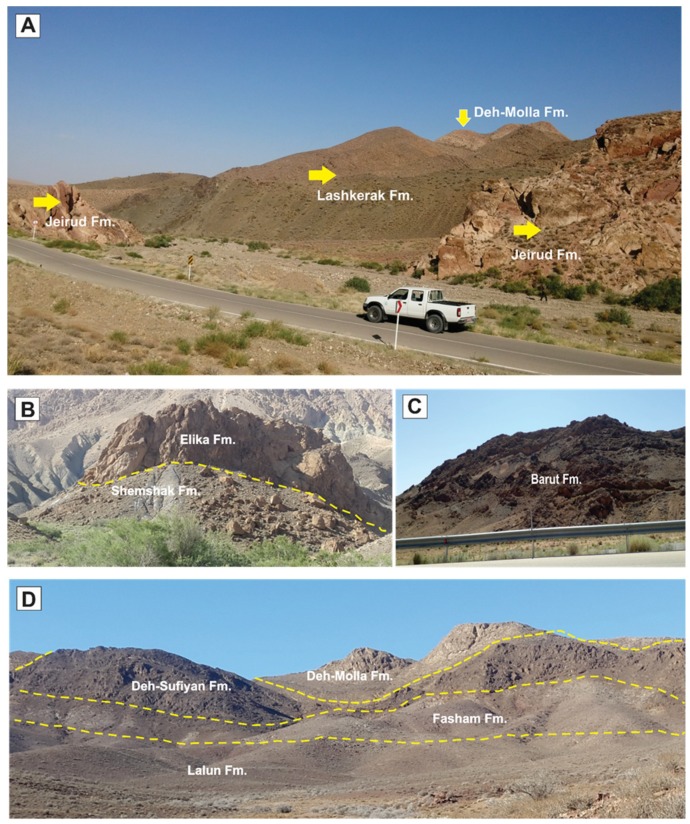
Outcrops of sedimentary lithological units in the Deh-Molla area. (**A**) Panoramic view of the Jeirud, Lashkarak, and Deh-Molla formations (Mila group); (**B**) carbonates of the Elika Formation showing fault contact with the Shemshak Formation; (**C**) dolomite and shales of the Barut Formation; and (**D**) panoramic view of the Lalun, Fasham, Deh-Sufyian and Deh-Molla (Mila group) Formations.

Fieldwork and laboratory analysis were carried out to identify the main petrographic and mineralogical characteristics of the sedimentary formations in the Deh-Molla area [27]. Sedimentological and stratigraphical fieldwork observations indicated that lithological units in the study area could be classified into three groups: (i) carbonates; (ii) clastics; and (iii) hybrids. Carbonate rocks are mainly composed of carbonate minerals such as calcite and dolomite. Clastic rocks include quartz, feldspars (potassium and plagioclase feldspars) and several accessory minerals such as clay minerals and mica. Hybrid rock units contain a mixture of carbonate and clastic rocks. Figure 3A–D shows some filed photographs of sedimentary formations exposed in the study area.

**Figure 4:**
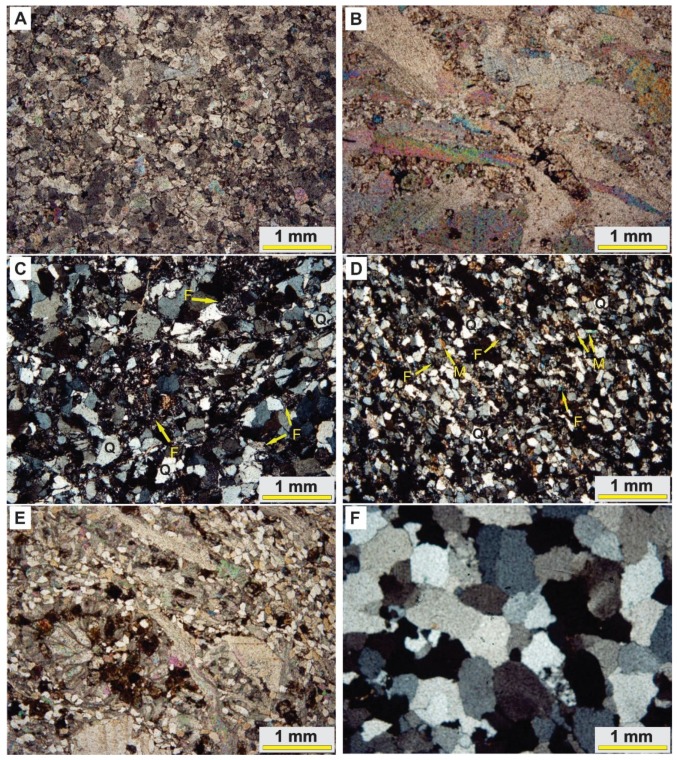
Photomicrographs of lithological units (rock thin sections under a petrographic microscope). (**A**) Soltanieh Formation showing high abundance of dolomite; (**B**) Deh-Sufyian limestone composed of calcite minerals and fossil shells; (**C**) arkosic sandstone of Lalun Formation composed of feldspars (F) and quartz (Q); (**D**) Jeirud Formation includes hybrid limestone with sandy quartz grains; (**E**) Deh-Molla Formations composed of quartz (Q) and feldspar (F) and few amounts of mica (M) with carbonates cement; and (**F**) quartz arenite sandstone of Fasham Formation mainly composed of quartz (Q).

**Figure 5:**
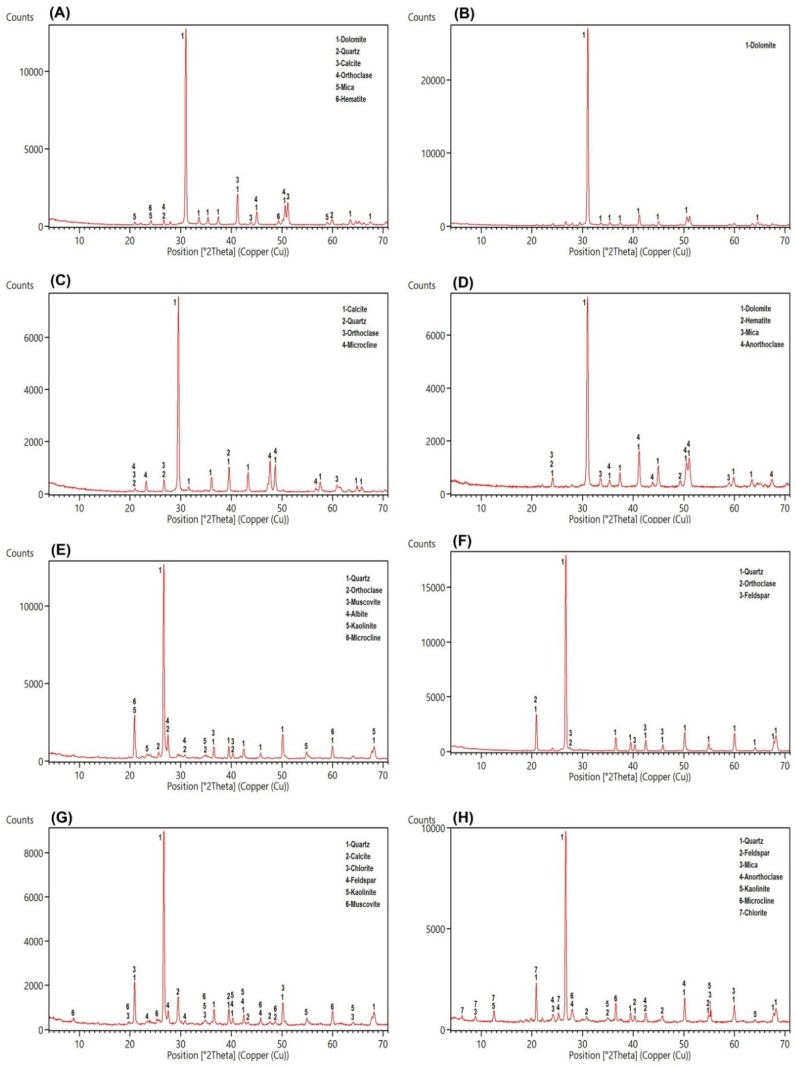

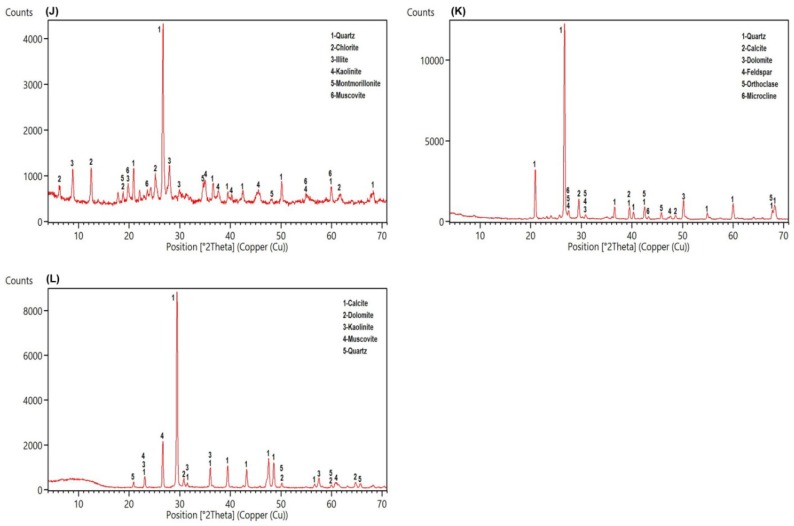
Results of XRD analysis shows minerals of representative samples collected from: (**A**) the Soltanieh Formation; (**B**) Bayandor Formation; (**C**) Deh-Sufiyan Formation (Mila group); (**D**) Elika Formation; (**E**) Lalun Formation (Mila group); (**F**) Fasham Formation (Mila group); (**G**) Lashkarak Formation (Mila group); (**H**) Shemshak Formation; (**J**) Barut Formation; (**K**) Deh-Molla Formation (Mila Group); and (**L**) Jeirud Formation.

Laboratory analysis was performed for extracting petrographic information from thin sections provided for representative samples of sedimentary formations. Moreover, detailed mineralogical information was obtained for the samples using XRD analysis. Soltanieh, Bayandor, Deh-Sufiyan (Mila Group) and Elika Formations are carbonate dominated lithological units in the Deh-Molla area. Figure 4A shows the microphotograph of the Soltanieh representative sample. Dolomite is dominated mineral in the thin section. It seems that brown to red color zones between dolomite crystals are iron oxide minerals due to alteration processes. The XRD analysis for the Soltanieh sample shows dolomite as major minerals and quartz, calcite, orthoclase, mica and hematite as accessory minerals (Figure 5A). The XRD result for the Bayandor representative sample displays only dolomite mineral (Figure 5B). However, petrographic information for Deh-Sufiyan Formation shows calcite and clastic deposits such as fossil shells (echinoderms and brachiopods) as well as quartz and feldspars (Figure 4B). Detected minerals by XRD for Deh-Sufiyan are calcite (major mineral), quartz, orthoclase and microcline (Figure 5C). The XRD analysis reveals the domination of dolomite in Elika with a minor amount of hematite, mica and anorthoclase (Figure 5D). 

Clastic rock units in the study area include Mila Group (Lalun, Fasham and Lashkarak) and Shemshak Formations (see Figure 3A–D). Microphotograph of the Lalun Formation displays that it composed of arkosic sandstone (Figure 4C). Quartz is the dominant mineral, and feldspar (mainly orthoclase, albite, and microcline) is also abundant (approximately 25%). In addition, small amounts of mica and clay minerals seem present (Figure 4C). Quartz, orthoclase, muscovite, albite, kaolinite and microcline were detected by XRD analysis (Figure 5E) in the Lalun representative sample. The Fasham Formation consist of mature quartzarenite sandstone (see Figure 4F), it has a white color feature that could be considered as the key bed during fieldwork. According to XRD analysis, it contains quartz as a major mineral with minor amounts of orthoclase and feldspar (Figure 5F). The Lashkarak Formation is an alternation of sandstones and shale (see Figure 3A). Quartz, calcite, chlorite, feldspars, kaolinite and muscovite were detected by XRD analysis in the Lashkarak representative sample (Figure 5G). The Shemshak Formation (see Figure 3B) is mainly composed of quartz, feldspars, mica, anorthoclase, kaolinite, microcline and chlorite based on XRD results (Figure 5H).

The Barut, Deh-Molla (Mila Group) and Jeirud Formations are considered as hybrid class in the study area. The Barut Formation consists of alternation of shale and dolomite beds (see Figure 3C). The XRD analysis results show the presence of quartz, chlorite, illite, kaolinite, montmorillonite and muscovite (Figure 5J). Microphotograph of a representative sample of the Deh-Molla Formation displays quartz, feldspar and few amounts of mica with carbonates cement (Figure 4E). According to XRD data, quartz, calcite, dolomite, feldspars, orthoclase and microcline are detected minerals for the Deh-Molla representative sample (Figure 5K). In the Jeirud Formation a mixture of carbonate and clastic deposits was identified (Figure 4D) in the microphotograph. In contrast with the Deh-Molla thin section (Figure 4E), calcite and dolomite are more dominated in Jeirud thin section (Figure 4D). Based on XRD analysis, calcite, dolomite, kaolinite, muscovite and quartz are dominated minerals, respectively (Figure 5L). Calcite and dolomite are major minerals, while kaolinite, muscovite and quartz are accessory minerals in the Jeirud representative sample.

### 4.2. BRMT Image Processing Results

**Figure 6:**
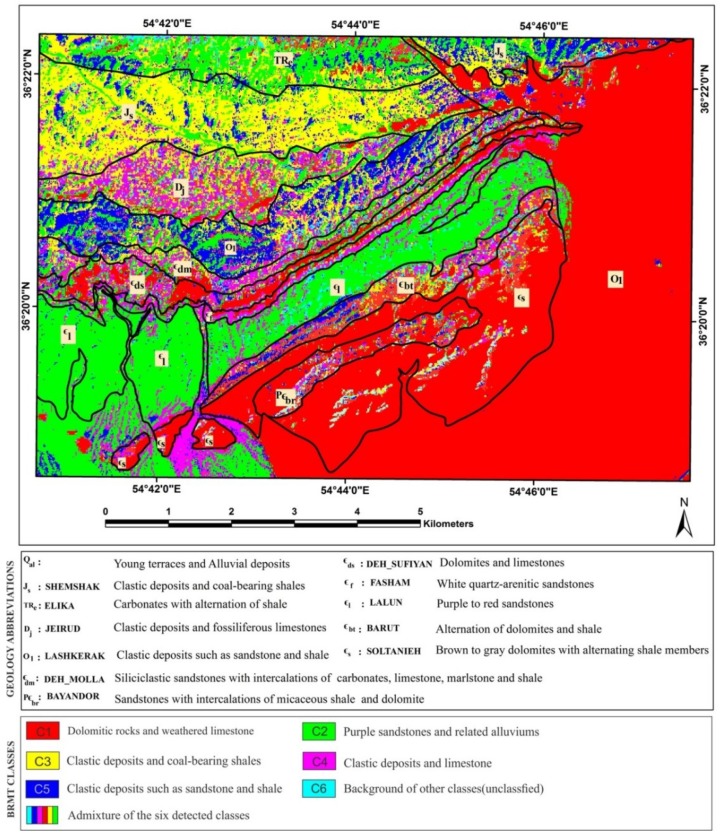
BRMT classification map of the Deh-Molla region.

Figure 6 shows resultant map derived from the post-classification (rule image classifier) of selected input BRMT bands for the Deh-Molla region. Sedimentary formations are discriminated based on specific spectral information extracted by BRMT approach. The resultant map shows six dominated classes (Figure 6). Color classes indicate different assemblages of minerals including quartz, calcite, dolomite, feldspar and mica. Red pixel class is generally governed in the study area especially in the southeastern and eastern parts. With reference to the geology map of the study area (see Figure 2), red pixel class (C1) is the Soltanieh and Bayandor Formations and Quaternary alluvium. These units mostly consist of carbonate rocks (dolomitic rocks) and weathered limestone. However, intercalations of sandstones and micaceous shale are also reported in the Soltanieh and Bayandor Formations, which are detected as pixels with different color classes in the red class background. 

Green pixel class (C2) in the central and southwestern parts of the scene (Figure 6) is the Lalun Formation, which is mainly composed of sandstones. However, green pixels are also mapped in several parts of the study area especially in association with Quaternary alluvium in the southwestern part of the study area. The yellow pixel class (C3) as the third dominated class is governed the central north and northwestern parts of the study area, where the Shemshak Formation is located. It contains clastic deposits and coal-bearing shales. Magenta pixel class (C4) mostly detected with the Jeirud Formation, which is composed of clastic deposits and limestone. However, the magenta pixel is also distributed in the Mila Formation, which is consisting of carbonates and siliciclastic sandstones with intercalations of carbonates, limestone, marlstone and shale. Moreover, this pixel class could be seen with Quaternary alluvium in the southwestern part of the scene (Figure 6). Blue pixel class (C5) is mainly mapped with the Lashkarak Formation. Clastic deposits such as sandstone and shale were reported in the Lashkarak Formation. However, blue pixels could also be found in the Shemshak, Elika, Barut Formations and many other parts of the study area. Cyan pixel class (C6) is not observed specifically with any sedimentary formation in the study area. This class shows less abundance and generally distributed in the background of other classes. The admixture of the six detected classes is mapped with the Barut, Elika and Zagun formations (Figure 6), which are composed of an alternation of carbonates and shale and clastic deposits. 

**Figure 7:**
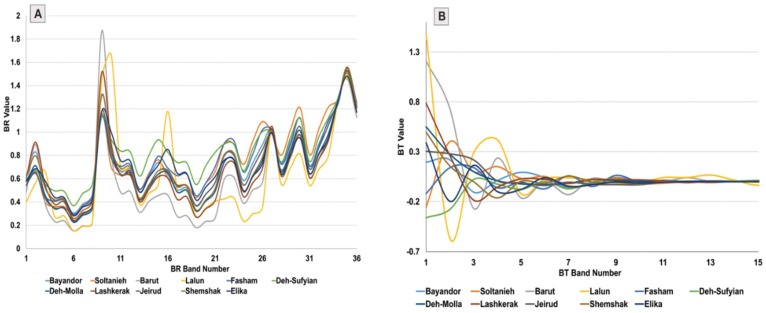
(**A**) Spectral signatures of the sedimentary formations in the study area extracted form band ratios (BR) matrix; (**B**) spectral signatures of the sedimentary formations after applying BRMT.

It is evident that similar spectral characteristics of sedimentary rocks influenced the results of BRMT in the classification map. Therefore, for finding the robustness of the BRMT, spectral signatures of the sedimentary formations in the study area compared with spectral properties extracted from BRMT (Figure 7A,B). High similarity in the spectral characteristics of sedimentary rocks is obvious in Figure 7A. Only the Soltanieh and Lalun Formations contain slightly different spectral signatures. It is probably due to their compositions with dominated dolomitic rocks (Soltanieh) and sandstones (Lalun). Spectral signatures of the sedimentary formations after applying BRMT display spectral differentiation between them especially from BT1 to BT3 (very strong). 

**Figure 8:**
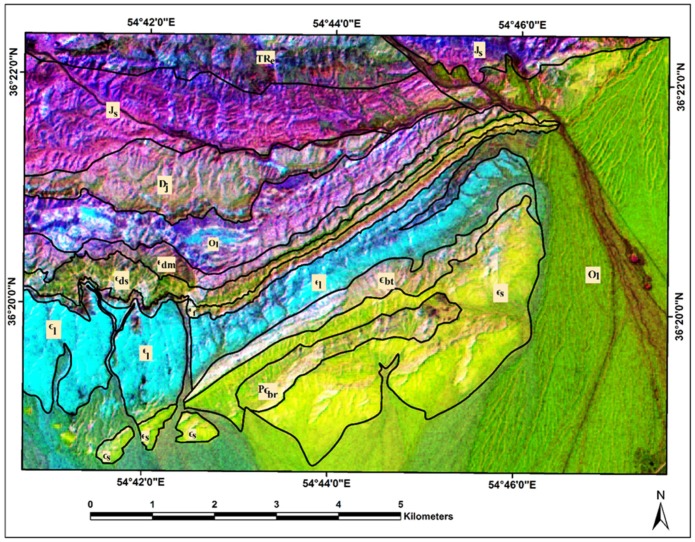
RGB Color composite of BRTM PC1, PC2, PC3 for the Deh-Molla region. Qal, Quaternary alluviums; Js, Shemshak Formation; TRe, Elika Formation; Dj, Jeirud Formation; Ol, Lashkarak Formation; Єdm, Deh-Molla Formation; Єds, De-Sufian Formation; Єf, Fasham; Єl, Lalun; Єz, Zagun Formation; Єbt, Barut Formation; Єs, Soltanieh Formation; PЄbr, Bayandor Formation.

Considering the statistical results, eigenvalues and %Vi of the forward BRMT for PC1–PC3 show the highest amount for them (see Table 1). As a result, they contain most spectral information in the image. These BRMT PCs (or BT bands) are assigned to RGB (Red, Green, and Blue) color composites for mapping sedimentary formations in the study area. Figure 8 shows the resultant image for Deh-Molla region. Comparing with the geology map of the study area, most of the sedimentary formations appear in recognizable tones. The Soltanieh, Bayandor, and Barut Formations and Quaternary alluvium are represented as yellow, greenish yellow, light orange and green colors, respectively (Figure 8). The Lalun and Zagun Formations manifested as cyan pixels with few blue and green pixels, which distributed in the boundary of these sedimentary formations. The Lashkarak, Jarud, Elika and Shemshak Formations appear as blue to purple color with some disseminations of cyan and blue pixels. The Mila Formations manifest mainly as mustard color. However, several mixing colors and variety of hues are observable in the background of sedimentary formations in the study area (Figure 8). With reference to the BRMT classification map of the Deh-Molla region, it is discernable that the BRMT PCs could not comprehensively identify the sedimentary formations compare to BRMT classification map (see Figure 6). Thus, it is required to consider other available statistical results for producing detailed color composites map of the sedimentary formations in the Deh-Molla region. 

**Figure 9:**
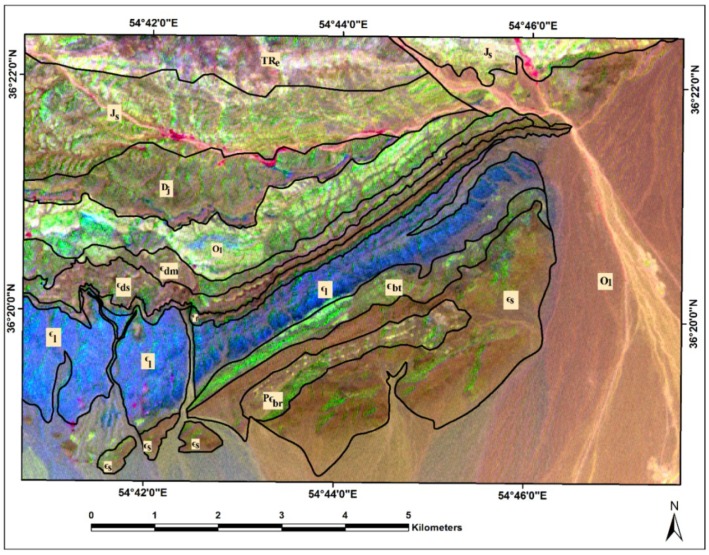
RGB color composite image map of most effective BRs as *n*1 = Red, *n*9 = Green and *n*32 = Blue for the Deh-Molla region. Qal, Quaternary alluviums; Js, Shemshak Formation; TRe, Elika Formation; Dj, Jeirud Formation; Ol, Lashkarak Formation; Єdm, Deh-Molla Formation; Єds, De-Sufian Formation; Єf, Fasham; Єl, Lalun; Єz, Zagun Formation; Єbt, Barut Formation; Єs, Soltanieh Formation; PЄbr, Bayandor Formation.

The deciphering of statistical results shows that Table 4 may contain some characteristics for finding the bands for high spectral differences. Table 4 shows the contribution percentage of 36 band ratios used for the construction of BRM. The BRs with high negative and positive contribution (C% > 3) are *n*1–*n*3, *n*9, *n*10, *n*21, *n*24–*n*28, *n*30, *n*31, and *n*33–*n*36. They contain visual information for considering that whether BRMT- based classification works properly or not. For producing RGB color composites, three BRs having strong contribution percentage were selected. Therefore, *n*1 (Positive C% = 5.408889 and Negative C% = 2.169569), *n*9 (Positive C% = 5.075753 and Negative C% = 1.716076), and *n*34 (Positive C% = 4.077037 and Negative C% = 3.132665) were used to assigning RGB color composites. Figure 9 shows a resultant map for the study area. Results indicate that most of the sedimentary formations were mapped more clearly compared to Figure 5; however, they match well with the BRMT classification map (see Figure 6). The resultant map was able to separate most of the sedimentary formations in the study area properly (Figure 9). The Lalun, Lashkarak, Jeirud, Elika, and Shemshak Formations are mapped and detected in differentiable tones. However, the Soltanieh, Bayandor, Barut, Zagun and Mila Formations and Quaternary alluvium appear in green to brown hues. 

The BRMT classification map of the Deh-Molla region (Figure 6) does not include topographical information. However, Figure 7, Figure 8 and Figure 9 contain valuable topographical and texture information. The most obvious texture in the study area that could be seen in the image maps is two sectors separated by a NE–SW line. The SE sector seems to expose a large sedimentary region of recent alluvial fans and minor topography, giving a fine-grained texture. The NW sector is sedimentary hard rocks in a stratigraphical series. Some of the blue pixels in Figure 6 present slope shade effects. The SE sector, gathering young alluvial sediments coming from Lar formation, is composed of limestone. Effects of color compositing have greatly helped to enhance spectral differences in the imagery. 

**Figure 10:**
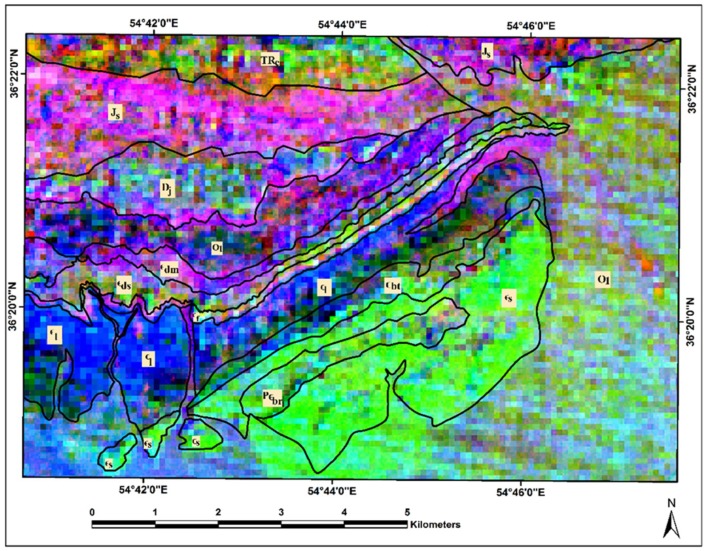
RGB color composite image map of KLI, CI, QI for the Deh-Molla region. Qal, Quaternary alluviums; Js, Shemshak Formation; TRe, Elika Formation; Dj, Jeirud Formation; Ol, Lashkarak Formation; Єdm, Deh-Molla Formation; Єds, De-Sufian Formation; Єf, Fasham; Єl, Lalun; Єz, Zagun Formation; Єbt, Barut Formation; Єs, Soltanieh Formation; PЄbr, Bayandor Formation.

**Figure 11:**
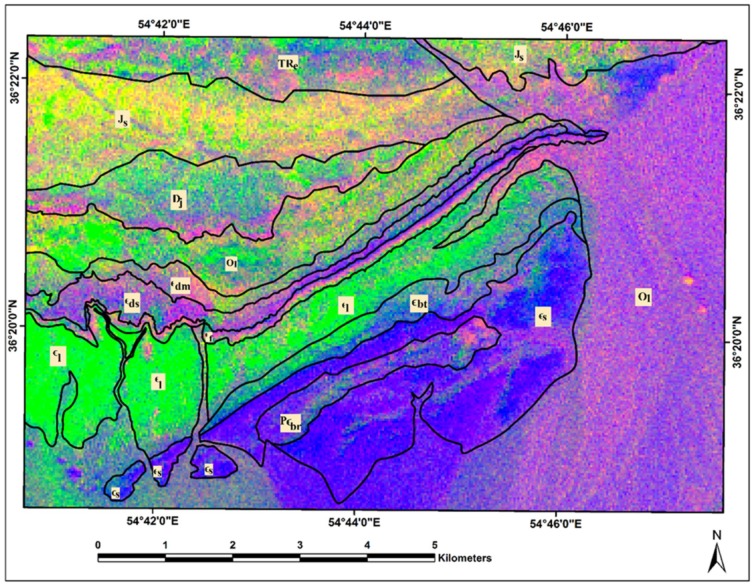
RGB color composite image map of OHI, ALI, DI for the Deh-Molla region. Qal, Quaternary alluviums; Js, Shemshak Formation; TRe, Elika Formation; Dj, Jeirud Formation; Ol, Lashkarak Formation; Єdm, Deh-Molla Formation; Єds, De-Sufian Formation; Єf, Fasham; Єl, Lalun; Єz, Zagun Formation; Єbt, Barut Formation; Єs, Soltanieh Formation; PЄbr, Bayandor Formation.

Considering the results achieved from the BRMT approach, it is evident that mapping sedimentary formation is a highly challenging task using ASTER spectral bands. However, this approach was able to extract appropriate information for mapping and discriminating sedimentary formations in the study region. Herein, the results of BRMT approach were compared with some well-established ASTER mineralogical indices such as OH-bearing-altered minerals Index (OHI) = (*b*7/*b*6 × *b*4/*b*6), Kaolinite Index (KLI) = (*b*4/*b*5 × *b*8/*b*6), Alunite Index (ALI) = (*b*7/*b*5 × *b*7/*b*8), Calcite Index (CI) = (*b*6/*b*8 × *b*9/*b*8), Dolomite Index (DI) = (*b*6 + *b*8)/*b*7) and Quartz Index (QI) = (*b*11/*b*10 × *b*11/*b*12) [12,14,18]. RGB color composite image map was produced by assigning Kaolinite Index (KLI), Calcite Index (CI) and Quartz Index (QI) for the study area (Figure 10). Moreover, OH-bearing-altered minerals Index (OHI), Alunite Index (ALI) and Dolomite Index (DI) were used to generate an RGB color composite image map (Figure 11). The resultant image maps indicate that the discrimination between the boundaries of sedimentary formations is very week using mineralogical indices. The pixels contain similar tones and colors almost are distributed in the background of all sedimentary formations in the study area (Figure 7 and Figure 8). The mineralogical–lithological indices were not capable of mapping and discriminating the sedimentary formations, including Soltanieh, Bayandor, Barut, Lalun, Mila Group, Jeirud, Shemshak and Elika, in the Deh-Molla region. 

## 5. Discussion

Mapping sedimentary rock is a very challenging task by application of conventional image processing techniques to satellite remote sensing data in a variety of geological environments [8,9,20,45,46]. Lithological and mineralogical indices proposed by Ninomiya and Fu [18] and Ninomiya et al. [12] for mapping sedimentary strata are threshold dependent and could not perform successfully in different geological regions. Furthermore, they may contain some wrong outputs as a result of the spectral interference of the minerals with similar absorption and reflection characteristics. Consequently, if only these indices are used for investigation, the lithological boundary would be mapped with low accuracy. The lack of specialized image processing method for mapping sedimentary rock units encouraged us to develop BRMT to map the sedimentary strata.

In this study, BRMT was developed and established for mapping sedimentary lithology using VNIR and SWIR ASTER satellite sensor spectral bands. In this approach, all forward and non-repetitive possible band ratios of VNIR and SWIR spectral bands of ASTER were considered for extracting the image spectra. Subsequently, the PCA was applied to the selected band ratios for enhancing spectral dissimilarities of the sedimentary rock units. Several mathematical equations have been developed in this analysis. The deciphering of statistical results indicates high potential of the BRMT for increasing the spectral differences among sedimentary lithologies. The BRMT was capable of mapping sedimentary succession in Deh-Molla region, North Iran. Most of the lithostratigraphic units, including Soltanieh, Bayandor, Barut, Lalun, Mila group, Shemshak and Jeirud were mapped and discriminated in the study area. Fieldwork data, petrographic study and XRD analysis revealed three groups of sedimentary rocks such as carbonates, clastics and hybrids in the Deh-Molla region. 

Petrographic study and XRD analysis emphasized the complexity of the sedimentary rock constituents and mineral assemblages in the study area. Dolomite, calcite and quartz with minor amounts of feldspars (mainly orthoclase, albite, and microcline) were the main constituents of the most of the sedimentary formation, which identified during the petrographic study. However, some alteration minerals (hematite, kaolinite and chlorite) were also in association with the main constituents of mineralogical content in representative samples of the sedimentary formations. Quartz, dolomite, calcite, orthoclase, microcline, anorthoclase, muscovite, albite, kaolinite, chlorite, montmorillonite and hematite were detected using XRD analysis. Therefore, the sedimentary formations exposed in the study area contain similar spectral characteristics with some small differences due to the variety of their mineralogical content. It indicates that the BRMT algorithm was able to reveal these small differences for geological mapping objectives. 

**Figure 12:**
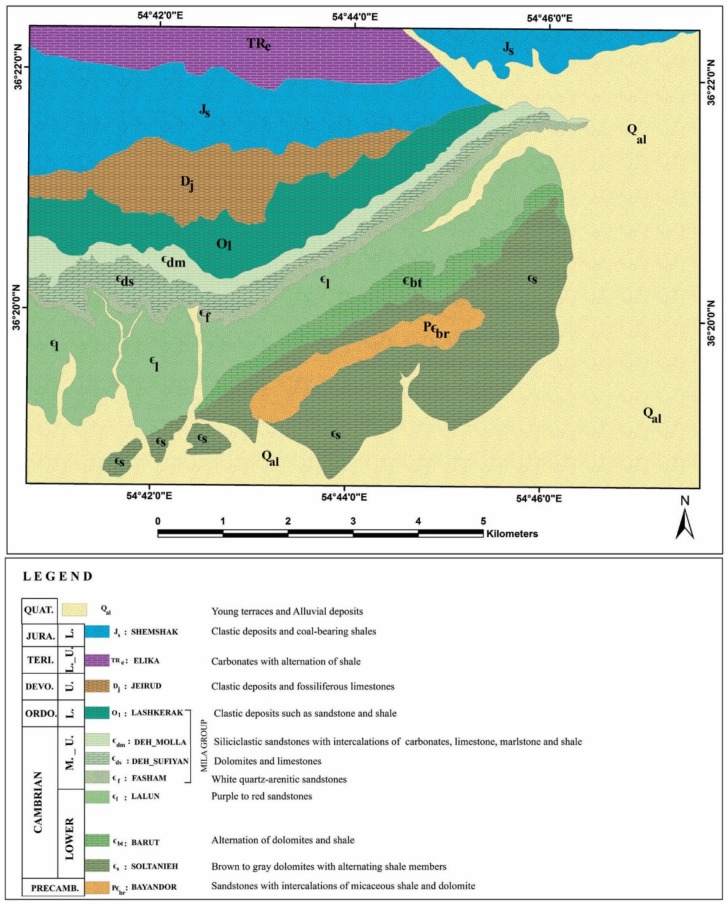
Geological map the Deh-Molla region derived from the BRMT approach.

Comparison of the results with the geological map of the study area, fieldwork and laboratory data indicate the robustness of the BRMT technique, despite similar constituents (mineralogical content) and spectral characteristics of sedimentary formations in the study area. Relative accuracy assessment was estimated for the BRMT classification map (Table 8). The overall accuracy of mapping results is 74.64% and the kappa coefficient is 0.70. It shows the robustness of the BRMT method, which was developed and established in this research. Figure 12 shows geological map that produced from the results of BRMT for the Deh-Molla region. It should be noted that Fasham Formation, introduced in this research, was not in the geological map of the study area, hence, corresponding accuracy assessment is not reported for Fasham Formation here.

## 6. Conclusions

The BRMT approach was developed in this study to characterize and discriminate the boundary of sedimentary rock formations in Deh-Molla region using VNIR and SWIR bands of ASTER remote sensing sensor. Sedimentary Formations exposed in the study region were discriminated and mapped based on specific spectral information extracted by BRMT approach. The results of fieldwork data and XRD analysis and petrographic information indicate three main sedimentary rock groups (carbonates, clastics and hybrids), which were successfully mapped by BRMT approach. Moreover, accuracy assessment of the mapping result indicates an appropriate kappa coefficient of 0.70 and appropriate overall accuracy of 74.64%. The results of this research demonstrated that the BRMT approach has great potential and capability for mapping sedimentary succession with diverse local–geological–physical characteristics in other regions around the world. 

## Figures and Tables

**Table 1:** Eigenvalues and %*V_i_* of the forward BRMT for *P*_1_–*P*_12_ for the selected subset of the study area.

*Pf_i_*	1	2	3	4	5	6	7	8	9	10	11	12
**Eigenvalue**	0.4488	0.1637	0.0396	0.0192	0.0085	0.0071	0.0038	0.0027	0.0024	0.0023	0.0005	0.0004
***%V_i_***	64.15	23.40	5.65	2.74	1.21	1.01	0.54	0.38	0.34	0.32	0.073	0.06

**Table 2:** Positive and negative correlation averages of band ratio candidates for components 1–12 in selected subset of the study area.

*p_k_*	1	2	3	4	5	6	7	8	9	10	11	12
$+{\bar{r}}_{k}$	0.40	0.20	0.13	0.27	0.24	0.17	0.19	0.20	0.19	0.30	-	-
$-{\bar{r}}_{k}$	0.78	0.59	0.28	0.23	0.15	0.26	0.13	0.12	0.49	0.12	-	0.12

**Table 3:** Positive and negative correlation of the band ratios *n*1–*n*36 in the construction of resultant principal components for a selected subset of the study area.

Band Ratio	Positive	Negative	Band Ratio	Positive	Negative
*n*1	0.36	−0.40	*n*19	0.00	−0.47
*n*2	0.21	−0.39	*n*20	0.00	−0.47
*n*3	0.24	−0.54	*n*21	0.00	−0.64
*n*4	0.11	−0.47	*n*22	0.19	−0.54
*n*5	0.14	−0.47	*n*23	0.21	−0.55
*n*6	0.08	−0.42	*n*24	0.22	−0.97
*n*7	0.10	−0.41	*n*25	0.20	−0.97
*n*8	0.16	−0.42	*n*26	0.20	−0.97
*n*9	0.33	−0.32	*n*27	0.59	−0.12
*n*10	0.37	−0.82	*n*28	0.23	−0.51
*n*11	0.04	−0.30	*n*29	0.17	−0.92
*n*12	0.14	−0.33	*n*30	0.22	−0.51
*n*13	0.05	−0.49	*n*31	0.23	−0.39
*n*14	0.11	−0.49	*n*32	0.18	−0.51
*n*15	0.05	−0.40	*n*33	0.20	−0.50
*n*16	0.16	−0.53	*n*34	0.27	−0.58
*n*17	0.12	−0.42	*n*35	0.31	−0.50
*n*18	0.11	−0.42	*n*36	0.28	−0.35

**Table 4:** Contribution percentage of band ratios *n*1–*n*36 in the construction of resultant principal components for a selected subset of the study area.

Band Ratio	Positive C%	Negative C%	Band Ratio	Positive C%	Negative C%
*n*1	5.408889	2.169569	*n*19	0	2.526033
*n*2	3.12804	2.091521	*n*20	0	2.521348
*n*3	3.698808	2.903388	*n*21	0	3.465243
*n*4	1.602283	2.554337	*n*22	2.851362	2.940772
*n*5	2.062588	2.557068	*n*23	3.215167	2.987117
*n*6	1.167184	2.274883	*n*24	3.306941	5.218326
*n*7	1.582557	2.216687	*n*25	2.983318	5.225227
*n*8	2.49382	2.259561	*n*26	3.119833	5.23804
*n*9	5.075753	1.716076	*n*27	8.997991	0.647781
*n*10	5.56376	4.453769	*n*28	3.438818	2.756674
*n*11	0.600233	1.610137	*n*29	2.569707	4.984671
*n*12	2.118782	1.789384	*n*30	3.385652	2.742368
*n*13	0.835992	2.624651	*n*31	3.54156	2.096501
*n*14	1.67863	2.619609	*n*32	2.785946	2.775086
*n*15	0.780654	2.181139	*n*33	3.020396	2.717943
*n*16	2.508453	2.862117	*n*34	4.077037	3.132665
*n*17	1.754094	2.248454	*n*35	4.697884	2.710994
*n*18	1.701826	2.247719	*n*36	4.320103	1.912448

**Table 5:** Band ratios with the correlation coefficient higher than 0.90 for a selected subset of the study area.

Band Ratio Number	Correlated Band Ratio	Band Ratio Number	Correlated Band Ratio
*n*6	*n*7, *n*8	*n*24	*n*22
*n*11	*n*12	*n*25	*n*22
*n*13	*n*6, *n*7, *n*8	*n*26	*n*22, *n*23, *n*24, *n*25
*n*14	n6,*n*7,*n*8	*n*28	n24, n25, n26
*n*15	*n*7, *n*8, *n*14	*n*29	*n*24, *n*25, *n*26, *n*28
*n*17	*n*13	*n*30	*n*24, *n*25, *n*26, *n*28, *n*29
*n*21	*n*7, *n*8, *n*13, *n*14, *n*15	*n*31	*n*24, *n*25, *n*26, *n*28, *n*29, *n*30
*n*19	*n*13, *n*14, *n*15, *n*17, *n*18	*n*32	*n*24, *n*25, *n*26, *n*28, *n*29, *n*30, *n*31
*n*20	*n*13, *n*14, *n*15, *n*17, *n*19, *n*20	*n*33	*n*24, *n*25, *n*26, *n*28, *n*30, *n*31, *n*33
*n*23	*n*22		

**Table 6:** BR bands with a specific manifestation (dark or bright pixels) of the sedimentary formations in the study area.

Band Ratio	Bright Formation	Dark Formation
*n*1		Lalun
*n*2	Lashkarak, Barut	
*n*3	Lalun	
*n*4, *n*5, *n*6, *n*7, *n*8	Alluvials	
*n*9		Deh-Molla, Jeirud
*n*10	Lalun	
*n*13, *n*14, *n*15	Alluvial	
*n*22	Lalun	
*n*23, *n*24, *n*25, *n*26	Alluvial	
*n*22, *n*23, *n*24, *n*25, *n*26	Soltanieh, Deh-sufyian	Lalun
*n*27		Fasham
*n*28, *n*29, *n*30, *n*31, *n*32, *n*33		
*n*35, *n*36	Fasham	

**Table 7:** Selected input BRMT bands with a specific manifestation (dark or bright pixels) of the sedimentary formations for post-classification.

BT Bands	Bright Formation	Dark Formation
*BT*1	Lalun	Deh-Sufyian, Deh-Molla, Alluvials
*BT*2		Lalun
*BT*3		Deh-Molla, Elika
*BT*4	Soltanieh, Lalun, Jeirud	Shemshak
*BT5*	Deh-Sufyian	
*BT6*	Soltanieh	
BT7	Fasham	
*BT13*	Lalun	Deh-Sufyian, Soltanieh
*BT18*	Barut	
*BT32*	Fasham	

**Table 8:** Relative accuracy estimated for lithological mapping results based on BRMT method via Shahrood geology map.

Formations	Prod. Acc.	User Acc.	Prod. Acc.	User Acc.
	(Percent)	(Percent)	(Pixels)	(Pixels)
**Elika**	98.63	60.54	3314/3360	3314/5474
**Shemshak**	75.1	70.71	8304/11057	8304/11744
**Jeirud**	59.88	89.3	4723/7887	4723/5289
**Lashkarak**	81.8	69.88	4388/5364	4388/6279
**Deh-Molla**	57	72.57	4092/7179	4092/5639
**Deh-Sufyian**	30	35.68	628/2093	628/1760
**Fasham**	-	-	-	-
**Lalun**	78.05	81.86	8632/11059	8632/10545
**Barut**	45.12	72.32	1915/4244	1915/2648
**Soltanieh**	77.19	55.23	5949/7707	5949/10771
**Bayandor**	66.42	59.71	1242/1870	1242/2080
**Alluvium**	85.51	83.92	26,057/30472	26057/31048
**Over. Acc.**	**Percent**		**Pixel**	
	74.64		70811/94860	
**Kappa Coef.**	**0.70**			
